# Protein-protein interactions of the nicotinamide/nicotinate mononucleotide adenylyltransferase of *Leishmania braziliensis*

**DOI:** 10.1590/0074-02760180506

**Published:** 2019-03-21

**Authors:** Lesly Ortiz-Joya, Luis Ernesto Contreras-Rodríguez, María Helena Ramírez-Hernández

**Affiliations:** Universidad Nacional de Colombia, Facultad de Ciencias, Laboratorio de Investigaciones Básicas en Bioquímica, Bogotá, Colombia

**Keywords:** Leishmania braziliensis, protein-protein interaction, Co-IP-MS/MS, NAD, NMNAT

## Abstract

**BACKGROUND:**

Nicotinamide adenine dinucleotide (NAD) plays a central role in energy metabolism and integrates cellular metabolism with signalling and gene expression. NAD biosynthesis depends on the enzyme nicotinamide/nicotinate mononucleotide adenylyltransferase (NMNAT; EC: 2.7.7.1/18), in which converge the *de novo* and salvage pathways.

**OBJECTIVE:**

The purpose of this study was to analyse the protein-protein interactions (PPI) of NMNAT of *Leishmania braziliensis* (LbNMNAT) in promastigotes.

**METHODS:**

Transgenic lines of *L*. *braziliensis* promastigotes were established by transfection with the pSP72αneoαLbNMNAT-GFP vector. Soluble protein extracts were prepared, co-immunoprecipitation assays were performed, and the co-immunoprecipitates were analysed by mass spectrometry. Furthermore, bioinformatics tools such as network analysis were applied to generate a PPI network.

**FINDINGS:**

Proteins involved in protein folding, redox homeostasis, and translation were found to interact with the LbNMNAT protein. The PPI network indicated enzymes of the nicotinate and nicotinamide metabolic routes, as well as RNA-binding proteins, the latter being the point of convergence between our experimental and computational results.

**MAIN CONCLUSION:**

We constructed a model of PPI of LbNMNAT and showed its association with proteins involved in various functions such as protein folding, redox homeostasis, translation, and NAD synthesis.

The cellular functions of proteins occur via concerted interactions with other proteins. Such protein-protein interactions (PPI) are the foundation of all biological processes occurring in different physiological and pathological conditions. They regulate several cellular functions, including cell cycle progression, signal transduction, metabolism, gene expression, vesicular transport, nuclear transport, and cell migration. Furthermore, members of the PPI network are potential therapeutic targets for the development of new drugs.^1^ Therefore, it is important to study and understand the specific nature of these interactions.

One such protein is the nicotinamide/nicotinate mononucleotide adenylyltransferase of *Leishmania braziliensis* (LbNMNAT), which is the key member in the biosynthesis of nicotinamide adenine dinucleotide (NAD) and has been functionally identified.^2^ PPI analysis by interaction networks has enabled the study of several biological aspects of human pathogens, such as the protozoan parasites *Leishmania* spp., *Plasmodium* spp., and *Trypanosoma* spp., which require the continuous discovery of pharmacological targets owing to their resistance mechanisms against existing drugs. In the case of *Leishmania*, PPI networks based on structural information have opened possibilities for further studies aimed at the development of novel drugs.^3^ These studies are crucial in the context of neglected diseases such as leishmaniasis, which is endemic to the tropical and subtropical regions of the world and causes approximately 30,000 deaths annually. Currently, there is no effective leishmaniasis vaccine available for humans.^4^ Therefore, it is necessary to explore the pathogenesis mechanisms of this parasite further, with focus on the identification of the PPI of key enzymes such as NMNAT. This offers a potential strategy for identifying and characterising new pharmacological targets. As proteins generally do not function in an isolated manner but are a part of a dynamic network, identifying the interaction partners of LbNMNAT can provide considerable information on their physiological function in the parasite.

Co-immunoprecipitation in combination with mass spectrometry (Co-IP-MS/MS) has been widely used for the sensitive and reliable identification of PPI and the characterisation of protein complex members.^5^ The interactions identified through the Co-IP-MS/MS strategy have been validated by other technologies, which reinforce the efficacy of MS to identify protein-protein associations.^6^

In this study, we aimed to investigate for the first time, the PPI of LbNMNAT by using experimental and bioinformatics tools such as Co-IP-MS/MS and PPI network construction, respectively. The identification of the members of the interaction network around LbNMNAT will expand the comprehension on its regulation in *L. braziliensis*. Furthermore, these regulatory members represent potential pharmacological targets to modulate NAD biosynthesis in the parasite.

## MATERIALS AND METHODS

*Culture of L. braziliensis promastigotes* - *L. braziliensis* (M2904 MHOM/BR/75M2904) promastigotes were cultivated in Schneider’s medium (pH 7.4; Sigma), supplemented with 10% (v/v) foetal bovine serum (FBS) at 26ºC, in 25-cm^2^ (T25) flasks. When the culture reached the stationary growth phase, the parasites were diluted to an initial concentration of 1 × 10^6^ cells/mL.

*Construction of the pSP72RαneoαLbNMNAT-GFP recombinant vector* - The fragment *lbnmnat* was polymerase chain reaction (PCR)-amplified using the plasmid pQE30-LbNMNAT as a template and the following oligonucleotides: forward 5ʹ-GGCTCTAGAATGTTATCCTCTACTGCT-3ʹ and reverse 5ʹ-GGCTCTAGAGGACGGAAGCCCCTC-3ʹ. The PCR product was cloned in the pGEMT-Easy (Promega) vector and was released from this vector employing the endonuclease XbaI (Fermentas) at 37ºC. The digested fragment was ligated with the expression vector pSP72RαneoαGFP^7^ (kindly donated by Dr Marcela Camacho), using the T4 ligase. The expression vector was previously digested with XbaI and dephosphorylated with alkaline phosphatase from calf intestine (Promega), for 2 h at 37ºC. The vector pSP72RαneoαLbNMNAT-GFP allowed the expression of the LbNMNAT enzyme fused with green fluorescent protein (GFP) in the C-terminus. The recombinant vector was verified by DNA sequencing.

*Transfection of L. braziliensis promastigotes* - The recombinant vectors pSP72RαneoαLbNMNAT-GFP and pSP72RαneoαGFP (empty) were used to transfect *L. braziliensis* promastigotes, following a standardised electroporation protocol.^7^ Briefly, parasites from 10 mL of a confluent culture were washed with 3 mL of cold Cytomix (25 mM HEPES pH 7.6, 120 mM KCl, 10 mM K_2_HPO_4_, 5 mM MgCl_2_, 2 mM EDTA, 0.15 mM CaCl_2_), centrifuged at 1000 *g* for 10 min at 4ºC. The precipitate of parasites was re-suspended in 1 mL of Cytomix and 450 μL of this suspension was transferred to a 4-mm electroporation cell containing 25 μg of the corresponding plasmids. The cell was incubated on ice for 10 min and 3 pulses of 1600 V, 25 μF capacitance and infinite resistance were applied, with intervals of 20 s between the pulses. The electroporated cells were incubated 24 h at 26ºC in a final volume of 5 mL of Schneider’s medium supplemented with 20% (v/v) FBS. Then, 5 mL of this medium supplemented with 120 μg/mL of geneticin G418 (Gibco-BRL) was added. The cultures were monitored daily by microscopic observations. Fifteen days later, live parasites were observed, and the established transgenic lines were cultured in the presence of 60 μg/mL G418. The transgenic parasites were used in immunodetection assays and fluorescence microscopy. GFP emission in paraformaldehyde-fixed cells was observed using the Nikon Eclipse C1 Plus microscope and the EZ-C1 software.

*Soluble protein extract preparation* - Transgenic parasites of each cell line were collected by centrifugation at 6000 *g* for 10 min at 4ºC, using 100 mL of the culture. The parasites were washed two times with 10 mL of PBS (pH 7.4), re-suspended in 500 µL of lysis buffer (0.1× PBS, cocktail of protease inhibitors (1:200; Sigma), 0.1% (v/v) Triton X-100) and incubated for 30 min at 4ºC with constant agitation. The suspension was centrifuged at 12000 *g* for 5 min at 4ºC and the supernatant was stored at -20ºC.

*Co-IP assays* - Soluble protein extracts (900 µL) were incubated with 100 µL of protein A-Sepharose (Sigma) for 1 h at 4ºC with constant agitation and centrifuged at 3000 *g* for 3 min at 4ºC. The anti-GFP antibody (Molecular Probes) was added to the clarified supernatant, to a final concentration of 0.2 µg/mL. The reaction was incubated for 16 h at 4ºC with agitation. Then, 100 µL of protein A-Sepharose was added and incubated for 2 h with agitation at 4ºC. The immunoprecipitate was obtained by centrifugation at 3000 *g* for 5 min at 4ºC. The precipitate was washed three times with washing buffer (50 mM Tris-HCl, pH 7.5, 150 mM NaCl, 5 mM MgCl_2_, 5% (v/v) glycerol). The immunoprecipitate was re-suspended in 100 µL of 1X sodium dodecyl sulfate polyacrylamide gel electrophoresis (SDS-PAGE) loading buffer (50 mM Tris-HCl, pH 6.8, 10% (v/v) glycerol, 2% (w/v) SDS, 0.1% (w/v) bromophenol blue, 100 mM β-mercaptoethanol) and heated at 92ºC for 10 min. The process was monitored by SDS-PAGE and western blot. For immunodetection, anti-GFP [1:500 in tris-buffered saline (TBS)] was used as the primary antibody, and peroxidase-conjugated anti-rabbit immunoglobulin G (IgG) (1:5000 in TBS; Sigma) was used as the secondary antibody. Membranes were revealed with 4-chloronaphthol (Promega) and hydrogen peroxide.

*Analysis of co-IP proteins by MS* - Here, 60 µL of the immunoprecipitates was analysed using T12 SDS-PAGE at 200 V for 5 min. Staining of the gel was completed with colloidal Coomassie G-250 (10% (w/v) (NH_4_)_2_SO_4_, 0.1% (w/v) Coomassie G-250, 3% (v/v) H_3_PO_4_, 20% (v/v) ethanol) for 16 h. The immunoprecipitated protein bands were extracted from the gel and identified by commercial nano-LC-MS/MS (https://www.alphalyse.com/). Samples were reduced with 150 μL 10 mM DTT in 50 mM NH_4_HCO_3_ and incubated for 30 min at 50ºC. Then, the samples were alkylated with 150 μL of 55 mM iodoacetamide in 50 mM NH_4_HCO_3_ and incubated for 30 min at room temperature, and digested with trypsin. The peptides were concentrated by lyophilisation and redissolved prior to being injected in a UPLC Dionex nano-LC system. The peptides were separated using a trap column (Acclaim PepMap 100, 75 µm × 2 cm) coupled to an analytic column (Acclaim PepMap RSLC, 75 µm × 15 cm) at 35ºC using eluent A (0.1% formic acid) and B (0.1% formic acid in 90% acetonitrile) with the following gradient: 10 min 5% B, 30 min 45% B, 5 min 98% B, 10 min 5% B; flow: 0.3 μL/min. MS/MS analysis was conducted in the Bruker Maxis Impact QTOF instrument using a CaptiveSpray with nanoBooster source (0.2 Bar), positive polarity mode, 50-2200 m/z mass range, 1500V capillary voltage, 3 L/min of dry gas, and 150ºC of dry temperature.

*Protein identification* - Proteomics data analysis was performed using the PatternLab for Proteomics software 4.0.^8^ Initially, the protein database of *L. braziliensis* (MHOM/BR/75/M2904) was loaded in the program from UniProt. As search parameters, we allowed two missed cleavages after trypsin digestion, precursor mass tolerance of 40 ppm, and carbamidomethylation (c) and methionine oxidation as fixed and variable modifications, respectively. Label-free quantification between common identified proteins in the biological replicates and the control sample was completed based on spectrum counting by means of the ratio: *Spectrum counts in a biological replicate/Spectrum counts in the control sample*.^9^ Fold change ≥ 1.5 was selected as the parameter.

*Identification of interactions of the LbNMNAT protein by network analysis* - The PPI network was created with the platform STRING V.10.5 (Search Tool for the Retrieval of Interacting Genes/Proteins).^10^ Resources used were neighbourhood, gene-fusion, text mining, co-expression, co-occurrence, and databases as well as experimental results, establishing a value of 0.4 as a minimum interaction score. Functional enhancement of the proteins was performed using functional classification systems in GO (Gene Ontology), and the functional and conserved modules were predicted using the databases InterPRO (https://www.ebi.ac.uk/interpro/), PFAM (https://pfam.xfam.org/), and KEGG (http://www.genome.jp/kegg/). Finally, the hypothetical proteins and interesting interactions were analysed. To contrast the results with experimentally confirmed physical PPI, a search was made for NMNAT physical interactions in *Homo sapiens*, *Escherichia coli*, *Saccharomyces cerevisiae*, and *Arabidopsis thaliana* in the online databases DIP (http://dip.doe-mbi.ucla.edu/dip/Main.cgi), BioGRID (https://thebiogrid.org/), and IntAct (https://www.ebi.ac.uk/intact/).

## RESULTS

**Fig. 1:**
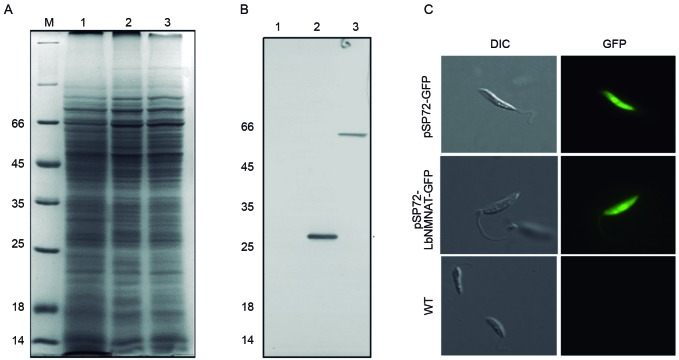
expression of the LbNMNAT-green fluorescent protein (GFP) in *Leishmania braziliensis* promastigotes*.* (A) Results from 12% sodium dodecyl sulfate polyacrylamide gel electrophoresis (SDS-PAGE) analysis and protein visualisation using Coomassie R-250. M, molecular weight marker (kDa). (B) The samples were transferred onto polyvinylidene difluoride (PVDF) membranes and immunodetected using the anti-GFP (1:1000) antibody. Lanes 1-3: soluble protein fraction of non-transfected, empty vector-transfected, and recombinant vector-transfected (pSP72RαneoαLbNMNAT-GFP) promastigotes, respectively. (C) Direct fluorescence was observed in the different cellular lines.

*LbNMNAT-GFP co-immunoprecipitates with diverse interaction partners* - Two transgenic cellular lines of promastigotes of *L. braziliensis* were established using the pSP72αneoαLbNMNAT-GFP and pSP72αneoαGFP vectors. Soluble protein extracts were obtained and analysed by western blot, whereas the parasites were observed by fluorescence microscopy to confirm LbNMNAT-GFP protein expression in the promastigotes. Parasites transfected with the empty vector expressed GFP (27 kDa), whereas those transfected with the recombinant pSP72αneoαLbNMNAT-GFP vector expressed the LbNMNAT enzyme fused to GFP (60 kDa) (Fig. 1).

**Fig. 2:**
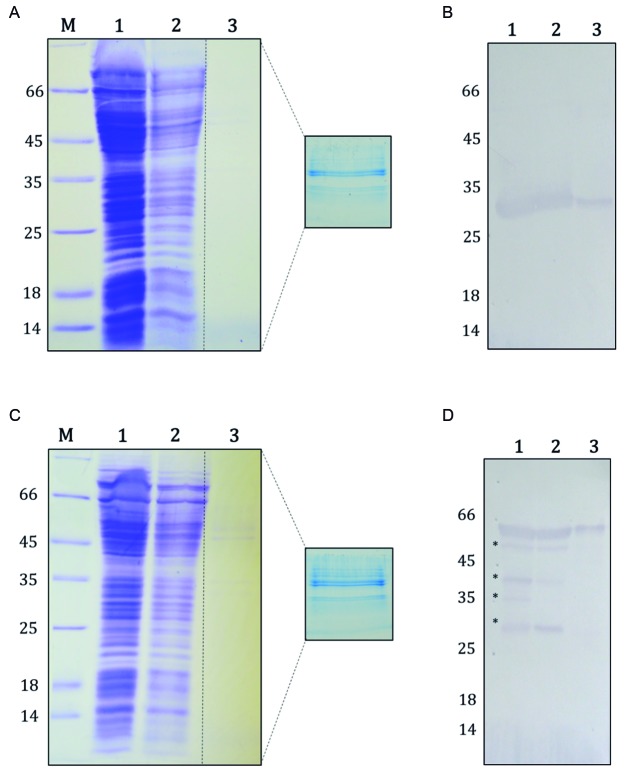
Co-immunoprecipitation of proteins associated with the LbNMNAT protein. (A and B) Co-immunoprecipitates from promastigotes expressing only green fluorescent protein (GFP). (C and D) Co-immunoprecipitates from promastigotes expressing recombinant LbNMNAT-GFP. (A and C) Results from 12% sodium dodecyl sulfate polyacrylamide gel electrophoresis (SDS-PAGE) analysis and proteins visualisation with colloidal Coomassie (G-250). The box samples, corresponding to the immunoprecipitates, were cut and analysed by Nano-LC-MS/MS. (B and D) Western blots obtained after using anti-GFP primary antibody (1:500) and anti-rabbit biotin secondary antibody (1:5000). 1. Clarified extract. 2. Post-immunoprecipitation soluble proteins. 3. Immunoprecipitates. The asterisks indicate possible products of processing or degradation of recombinant LbNMNAT-GFP.

Soluble protein extracts from parasites expressing LbNMNAT-GFP were used to perform co-immunoprecipitation experiments. To verify the dynamic nature of the PPI, two independent and complementary biological replicates were performed using asynchronous cellular cultures. To avoid unreliable positive results due to the co-immunoprecipitation of unspecified proteins, a parallel Co-IP assay was performed using soluble protein extracts from parasites that expressed GFP exclusively (control experiment). The obtained samples were analysed by SDS-PAGE and western blot. LbNMNAT-GFP was detected in each case, revealing that this protein can be immunoprecipitated adequately and specifically from soluble extracts (Fig. 2).

The co-immunoprecipitated proteins were analysed by nano-LC-MS/MS. Those common among the biological replicates 1 and 2 along with those common with the control sample but showing an upregulation of 1.5 fold or higher in the replicates are listed in Table I. We identified 103 possible interacting proteins, and the complete protein sets for all Co-IP-MS/MS experiments are listed in Supplementary data (Table). The putative functions of these proteins were obtained from the databases UniProt, GeneDB, and TriTrypDB.

**TABLE I:** Co-immunoprecipitation in combination with mass spectrometry (Co-IP-MS/MS) identified proteins in biological replicates 1 and 2

Protein	Uniprot access	Protein name	Score^*^	Biological replicate	Fold change^**^
1	A4H990	Nicotinamide mononucleotide adenylyltransferase	16.83	1 and 2	-
2	A4HC91	Putative 40S ribosomal protein S15	19.01	1 and 2	-
3	A4HHS1	Alpha tubulin	7.16	1 and 2	-
4	A4HGX9	Putative heat-shock protein hsp70 (Fragment)	6.71	1 and 2	-
5	A4HMZ0	Putative cystathione gamma lyase	7.99	1, 2 and control	1.5; 5.5
6	A4H727	Tubulin alpha chain	19.83	1, 2 and control	7; 8
7	A4H868	Putative 40S ribosomal protein S3	5.93	1, 2 and control	2; 3
8	A4HLE6	Putative 40S ribosomal protein S3	5.93	1, 2 and control	2; 3
9	A4HIH7	Putative heat shock 70-related protein 1,mitochondrial	20.50	1 and control	1.5
10	A4HIH9	Putative heat shock 70-related protein 1,mitochondrial	20.50	1 and control	1.5
11	A4HN57	T-complex protein 1 subunit	13.56	2 and control	1.7
12	E9AIH1	Contig, possible fusion of chromosomes 20 and 34	7.11	2 and control	1.5
13	A4HCU5	Putative 3-ketoacyl-CoA thiolase	5.27	2 and control	3
14	A4HCZ3	T-complex protein 1 subunit gamma	36.55	2 and control	6.5
15	A4HQL2	Putative T-complex protein 1, theta subunit	12.16	2 and control	3
16	A4HK82	T-complex protein 1 subunit epsilon	21.02	2 and control	2
17	A4HP03	Putative translation elongation factor 1-beta	13.35	2 and control	1.5
18	A4HLC9	Tubulin beta chain	23.32	2 and control	2.2
19	A4HLD6	Beta-tubulin	8.47	2 and control	2.5
20	A4HLD1	Beta-tubulin	14.89	2 and control	1.6
21	A4HC48	Tubulin beta chain	23.32	2 and control	2.2
22	A4HLC8	Tubulin beta chain	23.32	2 and control	2.2
23	A4HNM6	EF2-1 protein	8.84	2 and control	1.5
24	A4HPQ8	Adenosylhomocysteinase	11.21	2 and control	3
25	A4HPQ9	Adenosylhomocysteinase	11.21	2 and control	6
26	A4H7T5	ENOL protein (Fragment)	5.72	2 and control	2
27	A4H7T6	ENOL protein	21.78	2 and control	2.3

*: identification value assigned by PatternLab for Proteomics 4.0; **: label free quantification based on spectral counting. Proteins 5-8 present two values corresponding to fold changes between biological replicates 1 or 2 *vs* control sample, respectively.

**Fig. 3:**
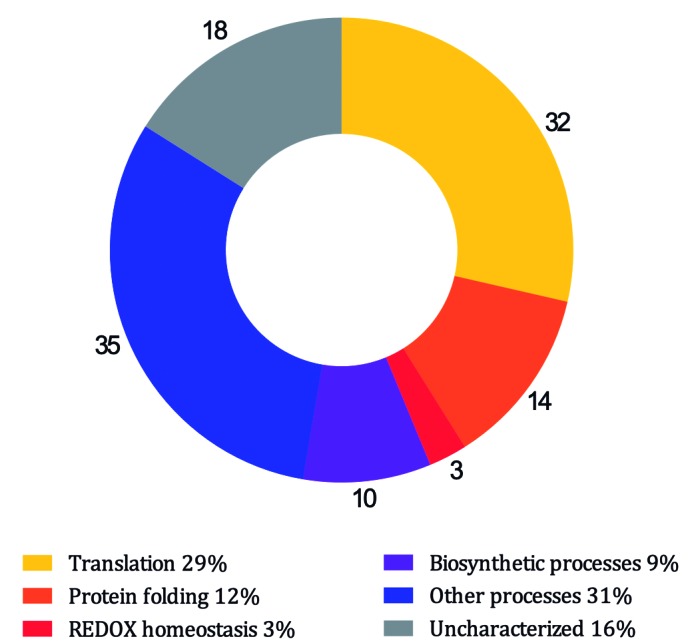
the proteins identified by co-immunoprecipitation and mass spectrometry (Co-IP-MS/MS) in the biological replicates 1 and 2 belong to diverse functional categories.

As mentioned, two independent Co-IP-MS/MS assays, using the soluble protein extracts from LbNMNAT-GFP-expressing parasites were performed. The proteins identified in each experiment were grouped and classified into six biochemical categories: proteins involved in translation, protein folding, redox homeostasis, biosynthetic processes, and other biological processes, and proteins with uncharacterised functions (Table II). The observed variation in the number of members per category could be attributed to the samples being obtained from asynchronous cellular cultures in which the promastigotes can exhibit relevant parasite development-related biochemical changes, which in some cases can modulate biological functions. Fig. 3 shows the functional category-based distribution of proteins identified using Co-IP-MS/MS in biological replicates 1 and 2 together.

**TABLE II:** Co-immunoprecipitation in combination with mass spectrometry (Co-IP-MS/MS) identified proteins classification into biochemical functions

Biological replicate 1		Biological replicate 2	
Biochemical function	Nº of proteins	Biochemical function	Nº of proteins
Translation	15	Translation	16
Protein folding	3	Protein folding	11
Biosynthetic processes	5	Redox homeostasis	3
Other processes	13	Biosynthetic processes	5
Uncharacterised	8	Other processes	23
		Uncharacterised	10

*Generation of a PPI network based on the LbNMNAT protein* - Several *in silico* methods have been developed to confirm the interactions detected experimentally. The computational methods for PPI prediction include approaches based on sequences, structures, chromosomal proximity, genetic fusion, *in silico* double hybrid, phylogenetic trees, gene ontology, and gene expression. Several web servers are available for the development of such analyses.^11^ In this study, we used an integrative approach for obtaining the physical and functional associations to create a PPI network; accordingly, we used the STRING web resource, which manages diverse sources of information facilitating a comparative analysis.^12^

**Fig. 4:**
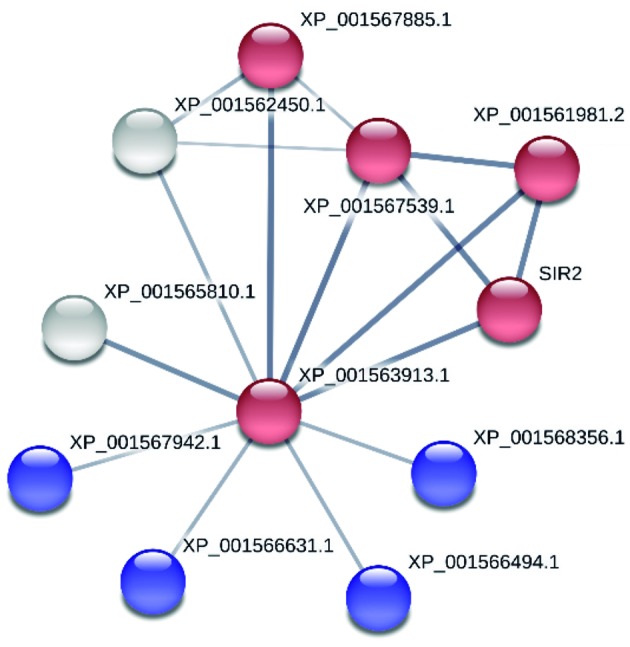
protein-protein interaction network of the LbNMNAT protein (STRING V.10.5). Reliable type view. The strongest associations are shown with thicker lines. Parameters: Score (0.4), no additional nodes; sources of interaction used: experimental, databases, co-expression, co-occurrence, gene fusion, and neighbourhood. LbNMNAT protein: node XP_001563913.1 (A4H990). Red: nodes associated with the metabolism of nicotinamide and nicotinate. Blue: nodes with RNA-binding motif. Grey: proteins not associated with cellular routes.

A PPI network for the LbNMNAT protein was generated (Fig. 4). The red node XP_001563913.1 (A4H990) represents the protein of interest and the remaining nodes represent the proteins interacting with LbNMNAT (Table III). The score for each node represents the confidence for each association and is derived by comparing the predictions and a reference set.^13^ During the creation of this network, it was arbitrarily established that the score of the minimal required interaction should be 0.4, employing all the sources of information except *textmining*. To validate the selected score, a PPI network based on the HsNMNAT1 protein was constructed, establishing two scores of minimal interaction: 0.9 (high) and 0.4 (medium). Identical results, in terms of number and identity of protein nodes, were obtained (data not shown). The physical interaction of HsNMNAT1 with RNA-binding proteins and sirtuins has been demonstrated,^14^ ^,^ ^15^ thus validating our bioinformatics approach.

**TABLE III:** List of predicted LbNMNAT interacting proteins according to STRING

Access^*^	Identifier (ID)^**^	Predicted functional partners	Score
A4H990	XP_001563913.1	LbNMNAT	-
A4HLF1	XP_001567885.1	Putative nicotinate phosphoribosyltransferase (413 aa)	0.964
A4HKF4	XP_001567539.1	Putative NAD synthase (293 aa)	0.964
A4H4L2	XP_001561981.2	ATP-NAD kinase-like protein (1257 aa)	0.911
E9AIR9	XP_003723185.1	Putative NAD-dependent SIR2 (284 aa)	0.909
A4HFG6	XP_001565810.1	Putative ribokinase (329 aa)	0.856
A4HF72	XP_001562450.1	Putative nitrilase (279 aa)	0.658
A4HMS6	XP_001568356.1	Putative RNA binding protein (458 aa)	0.629
A4HLK8	XP_001567942.1	Putative RNA-binding protein (129 aa)	0.629
A4HHU0	XP_001566631.1	Hypothetical protein (210 aa)	0.629
A4HHF3	XP_001566494.1	Putative RNA binding protein (648 aa)	0.629

*: access number in UniProt; **: access number in NCBI.

For LbNMNAT, we tested the same scores of minimal interaction (0.9 and 0.4) and detected 5 and 11 protein nodes, respectively. Considering that the obtained confidence scores were above 0.6 in the last case (0.4), the PPI network was constructed using this score of minimal interaction to present a more expanded network, which contains RNA-binding protein interactors, in accordance with our data obtained from Co-IP experiments. Among the parameters that describe the topology of the network, 11 nodes with a total of 16 connections, a 2.91 grade per node and a clustering coefficient of 0.885 were found.

The totality of the network proteins is predicted computationally from its nucleic acid sequence and has not been demonstrated experimentally. This can be attributed to the presence of high number of hypothetical proteins (~ 60%) in trypanosomatid genomes.^16^ Next, functional enhancement of the network was performed considering the information from the KEGG, PFAM, and INTERPRO databases, from which data on the biological routes and the predominant domains in the interacting proteins were collected. Five of the proteins, including NMNAT, are related to the metabolic route of nicotinate and nicotinamide, while four of the members exhibit RNA-binding motifs (Fig. 4), in concordance with our Co-IP-MS/MS experimental analysis.

## DISCUSSION

Proteins carry out their cellular functions via concerted interactions with other proteins, being PPI the basis of all biological processes. In this study, we found that proteins with diverse cellular functions interacted with LbNMNAT, and this can be explained by the cellular complexity, particularly the regulation of interactions in response to a particular signal, stimulus, or specific cellular state. Furthermore, they depend on many factors such as the stage of cellular development, the cell cycle phase, external conditions, and the presence of other proteins.^17^

*The LbNMNAT protein and its potential association with the ribosome* - In the functional classification of each set of co-immunoprecipitated samples, proteins mostly related to translation were found; 31 sequences are related to this process. Some of these are RNA-binding proteins or structural constituents of the ribosome. To validate their potential connection with LbNMNAT, interactions already characterised in others NMNATs of widely studied organisms were reviewed.

In *S. cerevisiae*, for example, NMNAT1 interacts physically with the RNA-binding proteins, MPT5 and HEK2 proteins.^18^ Human HsNMNAT1 has been found to interact with RBM4B, another RNA-binding protein which is also related to circadian regulation,^14^ and with RPL30 (ribosomal protein L30), a structural component of the ribosome. Furthermore, HsNMNAT1 interacts with STAU1, which is associated with double-stranded RNA and can play a role in the specific positioning of the mRNAs in specific areas of the cell.^14^ Additionally, through the affinity capture-MS technique, the interaction of HsNMNAT1 with the ribosomal proteins L11 (RPL11) and L22 (RPL22) was identified.^19^ These proteins are necessary for rRNA formation, maturation, and processing.

Currently, the significance of the interaction of NMNAT with the ribosome is unknown. Further research focusing on the stability of the complex and the interactions among their diverse components is essential because ribosome assembly and protein translation are finely coordinated with cellular growth, proliferation, differentiation, and development and because NAD is involved in all these processes.

*The LbNMNAT protein may participate in the folding of other proteins* - Among the co-immunoprecipitated proteins, 14 candidates were functionally related to the folding process. Cytoplasmic chaperones belonging to the T-complex and heat shock proteins (Hsp70, Hsp83, and Hsp60 chaperonin) were found. The *Leishmania* genome contains four genes encoding mitochondrial chaperonins: two of them, CPN60.2 and CPN10, are orthologs of the bacterial GroEL/GroES system. Pull down experiments for NMNATs in other organisms revealed that the homologous NadD of *E. coli* interacts with GroEL (Hsp60).^20^

Given the presence of T-complex proteins, a network of theoretical interactions was created using the software STRING. A network of high reliability (p-value: 5.67e-05) was obtained, with 10 nodes and 25 interactions (data not shown). This network predicts coordinated interactions among the different chaperonins of *L. braziliensis,* including the immunoprecipitated candidates. Potential interaction of LbNMNAT can be related to its protein-folding capacity, as has already been demonstrated in other NMNATs with chaperone functions independent of its NAD^-^synthesizing activity.^21^ This function can be considered as a response to stressful conditions, specifically drastic temperature changes, which is important for the survival of the parasite throughout its biological cycle.

The NMNAT-chaperone interaction has been demonstrated previously. In *H. sapiens*, HsNMNAT2 not only acts as a chaperone to reduce protein aggregates but also interacts with Hsp90 to retract them.^22^ Studies in *Drosophila melanogaster* have demonstrated that its NMNAT is necessary for thermal tolerance and responses against oxidative stress.^23^ Additionally, regulation of DmNMNAT and Hsp70 under stressful conditions has been shown to be considerably different at the transcriptional level, suggesting that the NMNAT, which exhibits constitutive expression, may represent a different class of stress-response proteins. Because it is a housekeeping protein, NMNAT is available under normal conditions and can provide a primary response to a stress condition, reducing the resulting proteotoxicity.^23^

*LbNMNAT may be involved in redox homeostasis* - The enzymes, gamma-glutamylcysteine synthetase 1 (GSH1) and tryparedoxin peroxidase (TXNPx), which are involved in redox homoeostasis, were also identified. Of these, TXNPx presents a greater potential for interaction with LbNMNAT.

The parasite can survive in the host owing to its redox metabolism, which counter arrests the radicals produced by infected macrophages. To avoid cellular damage by reactive oxygen species (ROS) and reactive nitrogen intermediates (RNI), trypanosomatids possess peroxidases that reveal a unique characteristic in the use of reducing equivalents derived from trypanothione. The combined action of TXNPx with antioxidant enzymes is crucial for the maintenance of a low concentration of H_2_O_2_, regulating the oxidative and nitrosative stress via the displacement of reducing equivalents from NADPH to hydroperoxides and peroxynitrites.^24^ Functional analysis has revealed that TXNPx is involved in the antimony resistance phenotype in *L. braziliensis*, suggesting that an increase in the expression levels of this enzyme plays a role in the clinical resistance to compounds derived from this element.^25^

*Leishmania* TXNPx has been studied previously,^24^ ^,^ ^25^ ^,^ ^26^ ^,^ ^27^ and it would be interesting to investigate whether a physical association exists among them. Co-IP assays have shown that the corresponding human homologues interact with each other.^28^ However, a direct interaction between these enzymes and the NMNAT protein has not been observed, and the interaction in homologues of other species has not been reported. Therefore, validation of a potential association through other experimental strategies is necessary, allowing the generation of new information in relation to metabolic routes and redox regulation under stressful conditions in the parasite.

*LbNMNAT PPI network* - Considering the PPI network, the proteins linked to the nicotinate/nicotinamide metabolism show a functional association. Among these proteins, putative nicotinate phosphoribosyltransferase (NaPRT) (XP_001567885.1) and putative NAD synthase (NADS) (XP_001567539.1) showed the highest scores. These enzymes belong to the salvage pathway of NAD synthesis. The substrate for NaPRT is nicotinic acid produced by nicotinamidase, which has been experimentally characterised.^29^ The product of the NaPR-catalysed reaction is nicotinic acid mononucleotide, the subsequent substrate of LbNMNAT. NADS catalyses the conversion of the LbNMNAT product, NAAD, into NAD, thus completing the salvage pathway. The three proteins (NaPRT, LbNMNAT, and NADS) act in series, which explains their high scores.

SIR2 in other *Leishmania* spp. has been studied previously,^30^ and the parasite is known to express three sirtuins: SIR2RP1, SIR2RP2, and SIR2RP3.^31^ After using the databases BioGRID and IntAct and identifying the homologues that interact in other species, it was found that human NMNAT1 physically interacts with SIRT1^15^ and NMNAT2 interacts with SIR4 in *S. cerevisiae*.^32^ Some authors have hypothesised that the close physical proximity of NMNAT1 and SIRT1 facilitate the most efficient use of NAD, probably through a substrate-channelling mechanism. Some mention that the enzymes involved in NAD biosynthesis can form a complex between this molecule and SIRT1, creating a micro-domain of high concentration of NAD that allows the regulation of sirtuin activity.^15^ Through direct validation, it would be interesting to study whether the physical interaction of NMNAT-SIR2 occurs in *Leishmania*.

We also found four RNA-binding proteins in the network. Their linkage was the product of relating homologous neighbour genes in other genomes (neighbourhood) and of the interaction of putative homologues in other species (*Campylobacter curvus*). The common characteristic of these proteins is that they all exhibit the RRM motif, found in several RNA-binding proteins. Our experimental results, in which 31 co-immunoprecipitated proteins (identified by MS) correspond to RNA-binding proteins, are in accordance with the constructed network. Furthermore, previous studies have also shown interactions between NMNATs and this type of RNA-binding proteins.

The Co-IP-MS/MS experiments and the STRING prediction showed a low overlap in terms of number and identity of the interacting proteins. This variation may be because the Co-IP-MS/MS experimental approach is based on sensitive instruments such as mass spectrometers, resulting in the high number of identified proteins in contrast with the STRING data. Although the Co-IP-MS/MS and bioinformatics data are not identical, both approaches indicated that the translation process is a common biochemical category to find the proteins interacting with LbNMNAT. Therefore, further studies are warranted to identify these interactors and investigate their physical interactions and to corroborate the results via other experimental techniques such as reciprocal pull down assays.

*In conclusion* - Using experimental and computational methods, we developed an integrated model of protein-protein interactions of the LbNMNAT enzyme. The model indicates the interaction of proteins related to a wide range of cellular activities, including protein folding, redox homeostasis, translation, and NAD synthesis. Our results expand the knowledge of NAD metabolism in *L. braziliensis* and offer new directions for scientific exploration, from an interactome point of view.
