# Supplementary material for: Protein-protein interactions of the nicotinamide/nicotinate mononucleotide adenylyltransferase of Leishmania braziliensis
Source: Mem Inst Oswaldo Cruz. 2019 Mar 21;114:e180506. doi: 10.1590/0074-02760180506 (PMC6430020; doi:10.1590/0074-02760180506)
Supplement: Supplementary file 1 [file 1678-8060-mioc-114-e180506-s.pdf]

TABLE

Co-immunoprecipitation in combination with mass spectrometry (Co-IP-MS/MS) identified proteins in biological replicates 1, 2 and control sample

Protein	Uniprot access	Protein name	Score*	Biological replicate
1	A4H990	Nicotinamide mononucleotide adenylyltransferase	16.83	1 and 2
2	A4HC91	Putative 40S ribosomal protein S15	19.01	1 and 2
3	A4HHS1	Alpha tubulin	7.16	1 and 2
4	A4HGX9	Putative heat-shock protein hsp70 (Fragment)	6.71	1 and 2
5	A4HMZ0	Putative cystathione gamma lyase	7.99	1, 2 and control
6	A4H727	Tubulin alpha chain	19.83	1, 2 and control
7	A4H868	Putative 40S ribosomal protein S3	5.93	1, 2 and control
8	A4HLE6	Putative 40S ribosomal protein S3	5.93	1, 2 and control
9	A4H5W0	Putative paraflagellar rod component	1.63	1
10	A4H9F9	Aconitate hydratase	5.81	1
11	A4H355	Putative acidocalcisomal exopolyphosphatase	1.71	1
12	A4HD17	Palmitoyltransferase	2.04	1
13	A4HAX5	Contig, possible fusion of chromosomes 20 and 34	3.62	1
14	A4HBR9	Putative 60S Ribosomal protein L36	5.09	1
15	A4H9U8	Putative seryl-tRNA synthetase	15.17	1
16	A4HEI3	Uncharacterised protein	2.86	1
17	A4H9Z7	Putative 40S ribosomal protein S13	9.57	1
18	A4HFK0	Putative small GTP-binding protein Rab1	12.73	1
19	A4H9H6	Uncharacterised protein	2.76	1
20	A4HIY0	Paraflagellar rod protein 1D	5.21	1
21	A4H3H6	D-3-phosphoglycerate dehydrogenase-like protein	4.51	1
22	A4H3Z5	Putative serine peptidase, Clan S-, family S54	2.64	1
23	A4HEU3	Putative 40S ribosomal protein S16	9.64	1
24	A4HK56	Putative RNA binding protein	28.70	1
25	A4HKU6	Uncharacterised protein	3.06	1
26	A4HFQ8	Putative arginyl-tRNA synthetase	2.65	1
27	A4HGT5	Eukaryotic translation initiation factor 3 subunit E	2.33	1
28	A4HAD7	Contig, possible fusion of chromosomes 20 and 34	5.13	1
29	A4HCF4	Uncharacterised protein	3.43	1
30	A4H679	Rab11 protein	3.07	1
31	A4H4D9	Dipeptidyl peptidase 3	3.35	1
32	A4HEM3	Putative 60S ribosomal protein L7	2.68	1
33	A4HEM2	Putative 60S ribosomal protein L7	2.68	1
34	A4H6P5	Putative 40S ribosomal protein S15A	9.26	1
35	A4HIY3	40S ribosomal protein S15a	9.26	1
36	A4HE62	Ribosomal protein S25	6.46	1
37	A4HAF8	Contig, possible fusion of chromosomes 20 and 34	6.46	1
38	A4HE61	ATP synthase subunit beta	16.50	1
39	A4HEJ4	ATPase beta subunit, putative (Fragment)	16.50	1
40	A4HFB9	Polyubiquitin, putative	10.20	1
41	A4HPM0	Putative polyubiquitin	10.20	1
42	E9AI36	Ubiquitin-fusion protein	10.20	1
43	A4HMK9	Putative 60S ribosomal protein L5	3.37	2
44	A4H5B1	Uncharacterised protein	7.22	2
45	A4H612	Isocitrate dehydrogenase [NADP]	2.30	2
46	A4HGX7	Activated protein kinase c receptor (LACK)	3.72	2
47	A4HC04	Putative RNA helicase	1.37	2
48	A4HG20	Putative heat shock protein DNAJ	8.43	2

↳

CC BY

Protein	Uniprot access	Protein name	Score*	Biological replicate
49	A4H9P0	Putative heat shock protein	2.30	2
50	E9AIQ3	T-complex protein 1 subunit delta	4.42	2
51	A4HPI7	14-3-3 protein-like protein	6.32	2
52	A4H4K3	Uncharacterised protein	1.29	2
53	A4H7A6	40S ribosomal protein S4	6.64	2
54	A4HAF4	Contig, possible fusion of chromosomes 20 and 34	1.97	2
55	A4HE10	Uncharacterised protein	8.72	2
56	A4HEE8	GTP-binding nuclear protein	4.24	2
57	A4HFA1	Uncharacterised protein	3.79	2
58	A4HKU9	Serine/threonine-protein phosphatase	5.59	2
59	A4HKK8	Uncharacterised protein	2.89	2
60	A4HM77	40S ribosomal protein S3a	1.92	2
61	A4H9R8	GSH1 protein	5.76	2
62	A4HLD5	Tubulin beta chain	8.43	2
63	A4HCU1	Acetyl-coenzyme A synthetase	2.52	2
64	A4HCR9	Acetyl-coenzyme A synthetase	2.52	2
65	A4H4K8	Putative 60S ribosomal protein L19	1.59	2
66	A4H4K5	Putative 60S ribosomal protein L19	1.59	2
67	A4H4K7	Putative 60S ribosomal protein L19	1.59	2
68	A4HP70	Chaperonin HSP60, mitochondrial	9.40	2
69	A4HP69	Chaperonin HSP60, mitochondrial	9.40	2
70	A4HFR5	60S acidic ribosomal protein P0	3.56	2
71	A4HFR6	60S acidic ribosomal protein P0	3.56	2
72	A4HG81	Katanin p60 ATPase-containing subunit A1	3.20	2
73	A4H3L6	Putative 26S protease regulatory subunit	3.20	2
74	A4HME8	Uncharacterised protein	3.20	2
75	A4H6T6	Putative proteasome regulatory ATPase subunit	6.67	2
76	A4HCA3	Putative proteasome regulatory ATPase subunit 1	3.20	2
77	A4HPV3	RPT6 protein	3.20	2
78	A4HGJ8	Putative ATPase	3.20	2
79	A4HGY0	Putative heat-shock protein hsp70 (Fragment)	19.96	2
80	A4H877	TRYP1 protein	2.29	2
81	A4H879	Tryparedoxin peroxidase	2.29	2
82	A4HMP1	Putative 60S ribosomal protein L12	2.94	2
83	A4HDT2	Putative 60S ribosomal protein L12	2.94	2
84	A4HC02	Putative 40S ribosomal protein S11	1.51	2
85	E9AIL3	Contig, possible fusion of chromosomes 20 and 34	1.51	2
86	A4HIH7	Putative heat shock 70-related protein 1,mitochondrial	20.50	1 and control
87	A4HIH9	Putative heat shock 70-related protein 1,mitochondrial	20.50	1 and control
88	A4HN57	T-complex protein 1 subunit	13.56	2 and control
89	E9AIH1	Contig, possible fusion of chromosomes 20 and 34	7.11	2 and control
90	A4HCU5	Putative 3-ketoacyl-CoA thiolase	5.27	2 and control
91	A4HCZ3	T-complex protein 1 subunit gamma	36.55	2 and control
92	A4HQL2	Putative T-complex protein 1, theta subunit	12.16	2 and control
93	A4HK82	T-complex protein 1 subunit epsilon	21.02	2 and control
94	A4HP03	Putative translation elongation factor 1-beta	13.35	2 and control
95	A4HLC9	Tubulin beta chain	23.32	2 and control
96	A4HLD6	Beta-tubulin	8.47	2 and control
97	A4HLD1	Beta-tubulin	14.89	2 and control
98	A4HC48	Tubulin beta chain	23.32	2 and control
99	A4HLC8	Tubulin beta chain	23.32	2 and control
100	A4HNM6	EF2-1 protein	8.84	2 and control

Protein	Uniprot access	Protein name	Score*	Biological replicate
101	A4HPQ8	Adenosylhomocysteinase	11.21	2 and control
102	A4HPQ9	Adenosylhomocysteinase	11.21	2 and control
103	A4H7T5	ENOL protein (Fragment)	5.72	2 and control
104	A4H7T6	ENOL protein	21.78	2 and control
105	A4HBC8	Contig, possible fusion of chromosomes 20 and 34	1.93	control
106	A4H377	Uncharacterised protein	2.24	control
107	A4HJB0	p-glycoprotein e	2.21	control
108	A4HJ93	Putative N-acyl-L-amino acid amidohydrolase	8.76	control
109	A4H8V4	Elongation factor 1-alpha	16.46	control
110	A4HA14	Uncharacterised protein (Fragment)	1.72	control
111	A4H3Q9	Uncharacterised protein	1.05	control
112	Q25225	Probable eukaryotic initiation factor 4A	11.58	control
113	A4HPQ1	Putative glycyl tRNA synthetase	1.89	control
114	A4H5F0	Stress-induced protein sti1	1.69	control
115	A4HII0	Putative heat shock 70-related protein 1,mitochondrial	31.97	control
116	A4HIH8	Putative heat shock 70-related protein 1,mitochondrial	27.85	control
117	A4HII6	Putative heat shock 70-related protein 1,mitochondrial (Fragment)	5.83	control
118	A4HII1	Putative heat shock 70-related protein 1,mitochondrial	5.83	control
119	A4HGY0	Putative heat-shock protein hsp70 (Fragment)	26.00	control
120	A4HGY1	Putative heat-shock protein hsp70	68.06	control
121	A4HLC8	Tubulin beta chain	10.02	control
122	A4HNM5	Elongation factor 2	1.54	control
123	A4HNM7	Elongation factor 2	17.07	control
124	A4HLL4	Putative elongation factor 1-gamma	4.06	control
125	A4H5S5	Elongation factor-1 gamma	56.70	control
126	A4HL71	HSP83 protein	22.65	control
127	A4HL69	HSP83 protein	22.65	control
128	A4HL70	Heat shock protein 83-1	30.30	control

*: identification value assigned by PatternLab for Proteomics 4.0.
